# Nontuberculous mycobacterial infections after cosmetic procedures: A case series

**DOI:** 10.1016/j.jdcr.2025.04.045

**Published:** 2025-07-29

**Authors:** Jenny Valverde-López, Percy Rojas Plasencia, Angie P. Mariños-Malón

**Affiliations:** Deparment of Dermatology, Hospital Regional Docente of Trujillo, Universidad Nacional of Trujillo, Trujillo, Peru

**Keywords:** cosmetic procedures, *Mycobacterium abscessus*, nontuberculous mycobacteria

## Introduction

In recent years, cosmetic procedures have gained significant popularity. By 2023, a 41.3% increase in cosmetic surgeries was reported over the previous 4 years, with liposuction and botulinic toxin injections being the most frequently performed procedures.^1^ Non-tuberculous mycobacteria (NTM) are acid-fast bacilli that differ from tuberculous mycobacteria in several aspects, including growth rate, temperature sensitivity, nutritional requirements, enzymatic activity, and resistance to antituberculous drugs.^2^

Clinical manifestations may vary, which makes diagnosis challenging and can delay appropriate treatment. Management typically involves a combination of macrolides and 1 or 2 additional antibiotics, depending on the extent of the infection and culture results; however, no clear clinical practice guidelines have been established.^3^ Furthermore, there are few reported cases of cutaneous NTM infections in Latin America, suggesting that the disease may be underdiagnosed and underestimated.

The following study is a case series of 7 patients without comorbidities who attended the outpatient service at Hospital Regional Docente in Trujillo, Peru. These patients presented with skin lesions following cosmetic procedures (Table I), from 2023 up to the date of consultation.

**Table I:** Demographics, clinical features, and laboratory results of the cases

Case	Gender/age (y)	Procedure and/or substance injected	Procedure performer and place of procedure	Incubation period	Lesion type	Localization	Treatment	Duration (mo)	Smear (microscopy)	Culture	Biopsy	PCR
1	F/53	DMAE	Cosmetologist/Beauty salon	60 d	2 nodules	Face	Minocycline 100 mg q24h + clarithromycin 500 mg q12h	8	+	*Mycobacterium sp.*	Dermatitis and panniculitis with lymphoplasmacytic predominance, and formation of few granulomas	M. abscessus
2	F/57	Tensor threads	General practitioner/Private practice	21 d	2 nodules	Face	Clarithromycin 500 mg q12h	6	+	*Mycobacterium sp.*	Acute and chronic unspecific inflammatory infiltrate with fat necrosis areas, and presence of a few granulomas	ND
3	F/34	Sodium bicarbonate	Cosmetologist/Spa	14 d	Multiple abscesses	Trunk	Doxycycline 100 mg q24h + clarithromycin 500 mg q24h	4	+	Negative	Chronic noncaseating granulomatous dermatitis	ND
4	F/40	Lipoabdominoplasty and lipotransfer	Plastic surgeon/Private practice	28 d	Multiple abscesses	Glutei	Sulfamethoxazole/trimethoprim 800/160 mg q12h + clarithromycin 500 mg q12h	10	+++	Negative	Noncaseating granulomatous inflammatory infiltrate	ND
5	F/30	Platelet rich plasma	General practitioner/Private practice	90 d	Infiltrated plaque	Left gluteus	Clarithromycin 500 mg q12h	4	+	*Mycobacterium sp.*	Granulomatous dermatitis	ND
6	F/42	Enzymatic hyaluronidase solution	General practitioner/Private practice	10 d	Erythematous nodules and fistulae	Abdomen	1st: Clarithromycin 500 mg q12h + Doxycycline 500 mg q24h + Levofloxacin 750 mg q24h 2do: Minocycline 100 mg q12h + clarithromycin 500 mg q12h	8 mo. Still under treatment.	+	Negative	Dense inflammatory infiltrate and histocytes forming granulomas	ND
7	F/23	Bichectomy and mentoplasty	General practitioner/Private practice	14 d	Erythematous nodules and fistula	Chin	Clarithromycin 500 mg q12h + doxycycline 100 mg q24h	6	+++	Negative	Chronic noncaseating granulomatous dermatitis	ND

*DMAE*, Dimethylaminoethanol; *ND*, not done; *PCR*, polymerase chain reaction.

### Case 1

**Fig 1:**
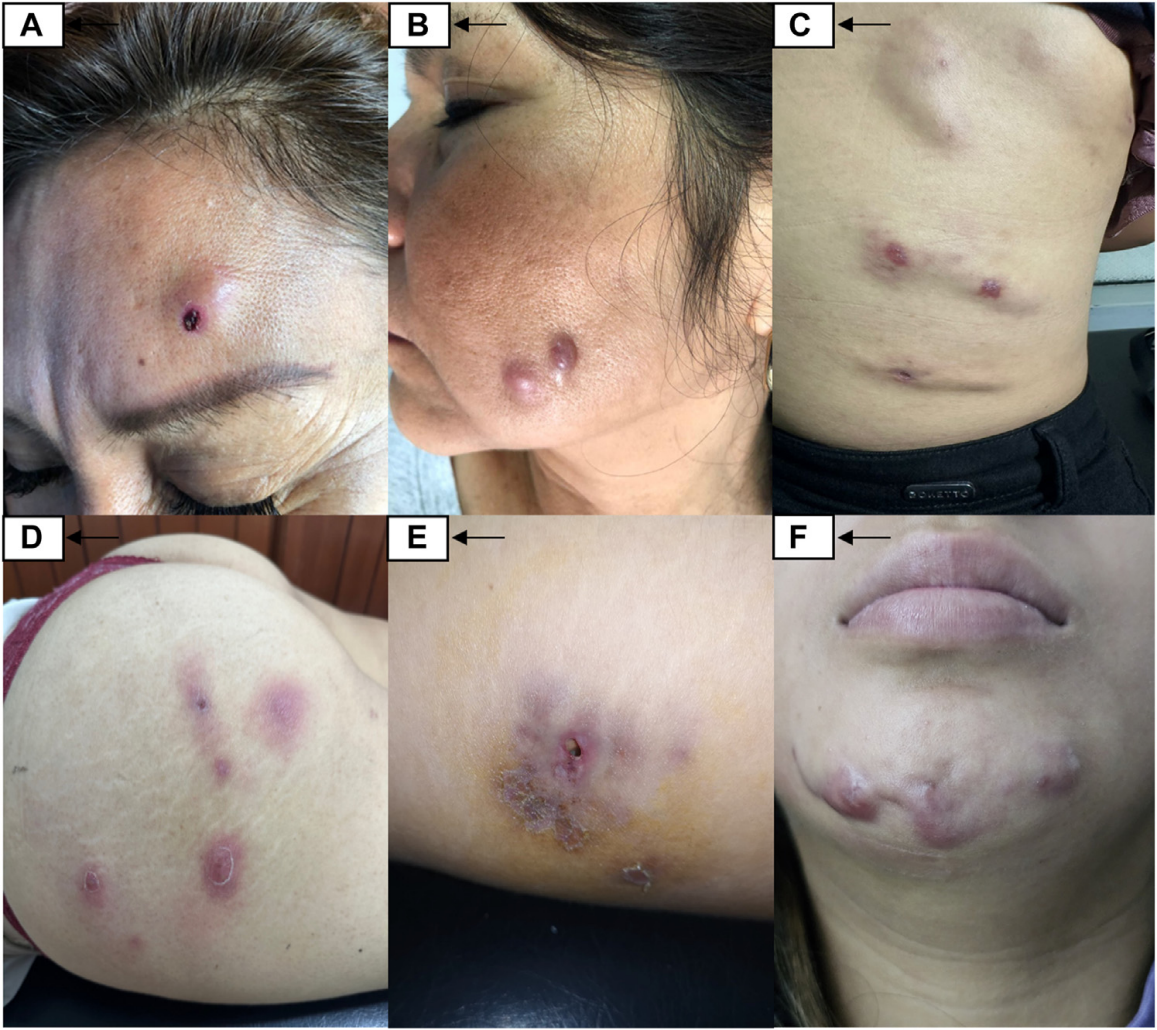
**A,** Nodules with raised borders and central ulceration, covered by a hematic scab on the frontal region of the face. **B,** Erythematous nodules in the left mandibular area. **C,** Multiple fluctuating abscesses on the posterior and lateral region of the trunk. **D,** Multiple fluctuating nodules and abscesses in gluteal region. **E,** Erythematous-violaceus plaque with a drainage opening on the left gluteus. **F,** Fluctuating nodules with a fistulous tract on the chin.

A 53-year-old female with no relevant medical history received intradermal infiltrations with dimethylaminoethanol (DMAE). Two months after the procedure, she developed nodular lesions at the injection sites on her face. She had previously received a 1-month course of antibiotics with no clinical improvement. On physical examination, erythematous nodules with central ulceration were observed in the frontal and left mandibular regions (Fig 1, *A*). Laboratory findings were unremarkable. Direct microscopy revealed acid-fast bacilli, and culture was positive for *Mycobacterium sp*. Skin biopsy demonstrated dermatitis and panniculitis with lymphoplasmacytic infiltrate and formation of granulomas. Ziehl-Neelsen (ZN) staining showed scant bacilli, which were identified by polymerase chain reaction (PCR) as *M. abscessus*. The patient was treated with minocycline, clarithromycin, and drainage, resulting in resolution of lesions.

### Case 2

A 57-year-old female with no relevant medical history, underwent a cosmetic procedure involving facial tensor threads. Three weeks post procedure, she developed nodular lesions at the punctures sites. She had previously received treatment with a cephalosporin, without clinical improvement. On physical examination, erythematous nodules were noted in the left mandibular region (Fig 1, *B*). Ultrasound revealed focal subcutaneous tissue thickening compatible with inflammatory edema. Microscopy demonstrated the presence of acid-fast bacilli, and culture was positive for Mycobacterium species. Skin biopsy revealed an acute and chronic nonspecific inflammatory infiltrate with areas of fat necrosis and the presence of a few granulomas. The patient was treated with clarithromycin and drainage, resulting in complete resolution.

### Case 3

A 34-year-old female, who received subcutaneous infiltrations with sodium bicarbonate on the posterior trunk developed painful nodules and purulent abscesses at the injection sites. She had previously undergone treatment with corticosteroids and antibiotics, without clinical improvement. On physical examination, multiple painful, fluctuant abscesses were observed on the posterior and lateral trunk (Fig 1, *C*). The culture was negative and the skin biopsy revealed chronic noncaseating granulomatous dermatitis. The patient was treated with clarithromycin and doxycycline, and clinical improvement was noted after 4 months. However, the patient was subsequently lost to follow-up.

### Case 4

A 40-year-old female, who underwent lipoabdominoplasty and gluteal lipotransfer developed skin lesions on site at the puncture sites 4 weeks after the procedure. She had been previously treated with cephalosporin and topical corticosteroid, with no clinical improvement. On examination, erythematous nodules and purulent abscesses were noted on both gluteal regions (Fig 1, *D*). Microscopy revealed numerous acid-fast bacilli. Skin biopsy demonstrated a noncaseating granulomatous inflammatory infiltrate in the superficial and mid-dermis, along with microabscesses in the fibroconnective and adipose tissue. The patient was treated with clarithromycin and sulfamethoxazole/trimethoprim and showed clinical improvement following treatment.

### Case 5

A 30-year-old female underwent through mesotherapy with platelet-rich plasma in the gluteal area and developed painful nodules at the injection site 3 months after the procedure. The patient had previously self-medicated with various topical antibiotics and intramuscular ceftriaxone without clinical improvement. Physical examination revealed an erythematous, indurated plaque with fluctuating areas and a draining sinus on the left gluteus (Fig 1, *E*). Soft tissue ultrasound showed a heterogenous fluid collection with internal echoes and a fistulous tract to the skin surface. Microscopy was positive for acid-fast bacilli and culture confirmed the presence of mycobacterium. Skin biopsy revealed granulomatous dermatitis. The patient was treated with clarithromycin, following which resulted in significant clinical improvement.

### Case 6

**Fig 2:**
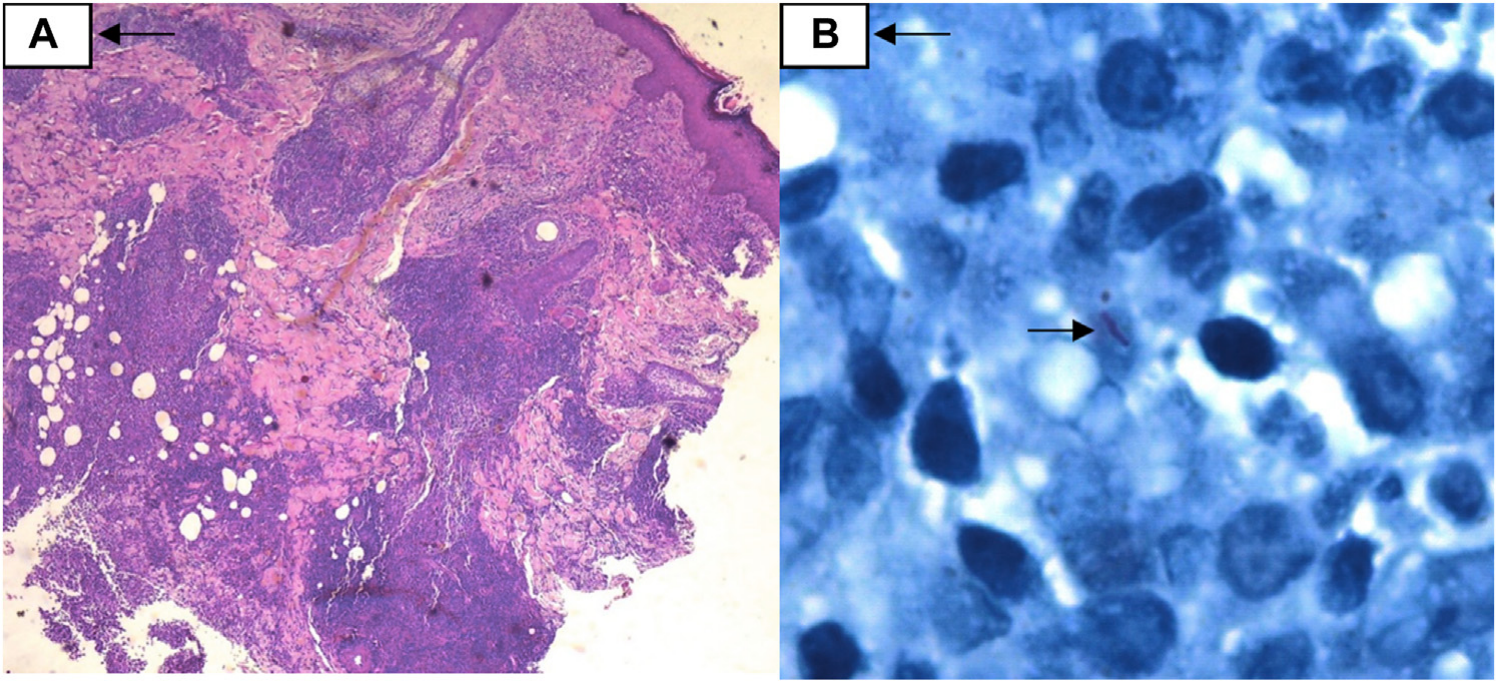
**A,** The epidermis shows acanthosis. From the superficial reticular dermis to the subcutaneous cellular tissue, there is a dense inflammatory infiltrate composed of lymphocytes, plasma cells, histiocytes and multinucleated giant cells forming granulomas. **B,** Ziehl-Neelsen staining positive for mycobacteria.

A 42-year-old healthy women developed abdominal abscesses 10 days after receiving subcutaneous injections of enzymatic hyaluronidase solution. The patient had been initially treated treated with antibiotics and abscess drainage without clinical improvement. On examination, multiple nodules, erythematous plaques, fistulous tracts, and scar lesions were observed. Microscopy evaluation revealed the presence of acid-fast bacilli. Skin biopsy showed a dense inflammatory infiltrate with histocytes forming granulomas (Fig 2, *A*). ZN staining confirmed the presence of bacilli (Fig 2, *B*). The patient was treated with clarithromycin, levofloxacin, doxycycline along with surgical drainage of the lesions. However, due to gastrointestinal intolerance, the antibiotic treatment was adjusted to minocycline and clarithromycin. The patient remains under antibiotic therapy, although multiple scar lesions were noted during the latest follow-up.

### Case 7

A 23-year-old female with no prior comorbidities, underwent bichectomy and mentoplasty with adipose tissue infiltration in the chin. Two weeks after the procedure, she developed skin lesions at the injection site. Initial management included drainage, antibiotic therapy but no clinical improvement observed. On physical examination, fluctuant erythematous nodules with a fistulous tract were identified in the chin region (Fig 1, *F*). Microscopy revealed numerous acid-fast bacilli. The culture was negative. Skin biopsy showed chronic noncaseating granulomatous dermatitis. The patient was treated with doxycycline and clarithromycin, with progressive clinical improvement documented during follow-up visits.

## Discussion

NTM infections have become a public health concern, making their diagnosis and proper treatment fundamentally important. Cutaneous NTM infection should be suspected in patients presenting with nodules and abscesses following cosmetic procedures, especially when there is no response to conventional antibacterial therapy.^4^ Rapid-growing mycobacteria typically associated with such cosmetic procedures include *M. abscessus, M. fortuitum, and M. chelonae*.^5^

Increase in the frequency of cosmetic procedures performed by both medical and nonmedical professionals has led to a decrease in quality standards and a neglect of basic aseptic requirements.^5^ Cosmetic procedures most commonly associated with cutaneous NTM infections include mesotherapy, dermal fillers, breast reconstruction, liposuction, CO_2_ laser resurfacing, body piercings, and tattoos. Other procedures, such as Mohs micrographic surgery and punch biopsies, have been highly implicated in the development of these infections as well.^6^ The procedures reported in this study included both surgical and nonsurgical interventions, performed by medical and nonmedical practitioners.

The clinical presentation is variable and depends on factors such as the duration of illness, the causative agent, and the patient’s immunologic status.^4^ Differential diagnoses include cutaneous tuberculosis, sporotrichosis, among others.^7^ Common clinical findings include abscesses, cellulitis, sporotrichoid nodules, ulcers, panniculitis, folliculitis, papules, and plaques with long-standing evolution, often accompanied by localized pain and possible fistula formation.^8^ Abscesses and nodules are the most frequent lesions observed in infections caused by *M. abscessus*, which were also the predominant findings in the patients described in this study.^9^

Microbial diagnosis is established through the isolation of the acid-fast bacilli using ZN staining, culture of skin tissue and/or drainage secretions, histopathological analysis and PCR. The gold standard remains culture, although it often requires prolonged incubation periods. In this study, NTM was isolated only through culture in 3 patients.^10^

PCR is considered the most sensitive diagnostic method, capable of identifying the microorganism in approximately 91% of the cases.^9^ However, in this study, PCR was performed in only one patient due to limited availability of the test in the city where the study was conducted. Histopathologic findings may vary and include suppurative, poorly formed tuberculoid, palisading or sarcoidal granulomas, typically accompanied by chronic inflammation.^6^

Antibiotic treatment, including drug selection, dosage and duration, depends on the severity and location of the infection, as well as the patient’s immune status. A multidisciplinary approach is always recommended.^4^ Currently, there are no standardized clinical practice guidelines for the treatment of cutaneous NTM infections. *M. abscessus* is notably resistant to many antibiotics, with limited susceptibility to agents such as imipenem. It may be sensitive to clarithromycin and amikacin, although resistance can occur, particularly in the presence of the erm gene.^11^ Monotherapy with clarithromycin has shown success in treating *M. chelonae*; however, to reduce the risk of resistance, a combination of at least 2 antibiotics is advised for infections caused by rapidly growing mycobacteria.^12^ Accordingly, treatment for the patients in this study included macrolides in combination with quinolones and tetracyclines, following current therapeutic recommendations.

In conclusion, rapidly growing mycobacteria infections are not classified as notifiable diseases, which contributes to a lack of awareness and underestimation of their true prevalence in this country. It is essential that public health authorities address the current epidemiological landscape to identify potential sources of transmission and implement preventive measures. Moreover, further research is needed to better understand the morbidity and mortality associated with these infections and to establish comprehensive antibiotic susceptibility profiles. Given that treatment regimens are prolonged, involve multiple high-dose antibiotics, and often result in permanent scarring, the development of standardized clinical practice guidelines is crucial for effective and timely management.^2^

## Conflicts of interest

None disclosed.
